# The effects of topical mesenchymal stem cell transplantation in canine experimental cutaneous wounds

**DOI:** 10.1111/vde.12011

**Published:** 2013-02-22

**Authors:** Ju-Won Kim, Jong-Hwan Lee, Young S Lyoo, Dong-In Jung, Hee-Myung Park

**Affiliations:** * Department of Veterinary Internal Medicine, College of Veterinary Medicine, Konkuk UniversitySeoul, South Korea; † Department of Veterinary Anatomy, College of Veterinary Medicine, Konkuk UniversitySeoul, South Korea; ‡ Department of Veterinary Pathology, College of Veterinary Medicine, Konkuk UniversitySeoul, South Korea; § Department of Veterinary Internal Medicine, College of Veterinary Medicine, Gyeongsang National UniversityJinju, South Korea

## Abstract

**Background:**

Adult stem cells have been widely investigated in bioengineering approaches for tissue repair therapy. We evaluated the clinical value and safety of the application of cultured bone marrow-derived allogenic mesenchymal stem cells (MSCs) for treating skin wounds in a canine model.

**Hypothesis:**

Topical allogenic MSC transplantation can accelerate the closure of experimental full-thickness cutaneous wounds and attenuate local inflammation.

**Animals:**

Adult healthy beagle dogs (*n* = 10; 3–6 years old; 7.2–13.1 kg) were studied.

**Methods:**

Full-thickness skin wounds were created on the dorsum of healthy beagles, and allogenic MSCs were injected intradermally. The rate of wound closure and the degree of collagen production were analysed histologically using haematoxylin and eosin staining and trichrome staining. The degree of cellular proliferation and angiogenesis was evaluated by immunocytochemistry using proliferating cell nuclear antigen-, vimentin- and α-smooth muscle actin-specific antibodies. Local mRNA expression levels of interleukin-2, interferon-γ, basic fibroblast growth factor and matrix metalloproteinase-2 were evaluated by RT-PCR.

**Results:**

Compared with the vehicle-treated wounds, MSC-treated wounds showed more rapid wound closure and increased collagen synthesis, cellular proliferation and angiogenesis. Moreover, MSC-treated wounds showed decreased expression of pro-inflammatory cytokines (interleukin-2 and interferon-γ) and wound healing-related factors (basic fibroblast growth factor and matrix metalloproteinase-2).

**Conclusion and clinical importance:**

Topical transplantation of MSCs results in paracrine effects on cellular proliferation and angiogenesis, as well as modulation of local mRNA expression of several factors related to cutaneous wound healing.

**Résumé:**

**Contexte**

Les cellules souches adultes ont été largement étudiées dans les approches de bio-ingénierie pour la thérapie de réparation tissulaire. Nous évaluons l'efficacité clinique et la sécurité de l'application de cellules souches mésenchymateuses allogéniques en culture dérivées de moelle osseuse (MSCs) pour le traitement de plaies cutanées dans un modèle canin.

**Hypothèse**

La transplantation de MSC allogénique topique peut accélérer la fermeture en toute épaisseur de plaies cutanées expérimentales et atténuer l'inflammation locale.

**Sujets**

Des chiens beagles adultes sains (*n* = 10; 3–6 ans; 7.2–13.1 kg) ont été étudiés.

**Méthodes**

Des plaies cutanées en pleine épaisseur ont été crées sur la face dorsale des beagles sains et des MSCs allogènes ont été injectées par voie intradermique. Le taux de cicatrisation et le degré de production de collagène ont été analysés sur le plan histologique par colorations à l'hématoxyline et éosine et par trichrome. Le degré de prolifération cellulaire et d'angiogénèse ont été évalués par immunohistochimie à l'aide d'anticorps spécifiques d'antigène nucléaire de prolifération cellulaire, de vimentine et d'actine de muscle lisse α. Les taux d'expression local d'ARNm d'interleukine-2, d'interféron-γ, du facteur de croissance basique de fibroblaste et de métalloprotéinase-2 de matrice, ont été évalués par RT-PCR.

**Résultats**

Comparé avec les plaies traitées par excipient, les plaies traitées par les MSCs ont montré une plus rapide cicatrisation et une augmentation de synthèse de collagène, de prolifération cellulaire er d'angiogénèse. En outre, Les plaies traitées au MSC ont montré une expression diminuée de cytokines pro-inflammatoires (interleukine-2 et interféron-γ) et des facteurs liés à la cicatrisation (facteur de croissance basique de fibroblaste et métalloprotéinase-2 de matrice).

**Conclusion et importance clinique**

La transplantation topique de MSCs résulte en des effets paracrines de prolifération cellulaire et d'angiogénèse ainsi qu'en une modulation de l'expression locale d'ARNm de plusieurs facteurs liés à la cicatrisation cutanée.

**Resumen:**

**Introducción**

Las células madre de adultos se han estudiado extensivamente en estrategias de bioingeniería para la reparación de tejidos. Hemos evaluado el valor clínico y la seguridad de la aplicación de células madre mesenquimales (MSCs) alogenéicas derivadas de la medula ósea para tratar las heridas de la piel en un modelo canino.

**Hipótesis**

El transplante tópico alogenéico de MSCs puede acelerar la cicatrización de heridas cutáneas experimentales de todo el grosor de la piel y disminuir la inflamación local.

**Animales**

Se estudiaron perros de raza Beagle sanos adultos (n=10; de 3-6 años de edad; 7,2-13,1 kg de peso).

**Métodos**

Se crearon heridas de todo el grosor de la piel en el dorso de perros Beagle sanos, y se inyectaron MSCs alogenéicas intradérmicamente. El ritmo de reparación de la herida y el grado de producción de colágeno se analizaron histológicamente utilizando tinciones de hematoxilina-eosina y tricrómico. El grado de proliferación celular y la angiogenesis se evaluaron mediante inmunohistoquímica utilizando anticuerpos específicos para el antígeno de proliferación celular nuclear (PCNA), vimentina y α-actina de músculo liso. La expresión local de mRNA para interleuquina-2 (IL-2), interferón- γ (IFN- γ), factor básico de crecimiento de fibroblastos (BFGF) y metaloproteinasa de matriz-2 se evaluaron mediante RT-PCR.

**Resultados**

comparado con las heridas tratadas solo con el vehículo, las heridas tratadas con MSCs mostraron una reparación más rápida y un aumento en la producción de colágeno, proliferación celular y angiogenesis. Además, las heridas tratadas con MSCs mostraron una expresión aumentada de citoquinas proinflamatorias (IL-2 e IFN- γ) y factores relacionados con la reparación de la herida (BFGF y metaloproteinasa de matriz-2).

**Conclusión e importancia clínica**

El transplante tópico de MSCs resulta en efectos paracrinos en la proliferación celular y angiogenesis, así como en la modulación de la expresión de mRNA de diversos factores relacionados con la cicatrización de heridas.

**Zusammenfassung:**

**Hintergrund**

Adulte Stammzellen sind für ihre biotechnologische Verwendung bei der Wiederherstellung von Geweben bereits weit reichend erforscht. Wir haben die klinische Bedeutung und Sicherheit der Applikation von kultivierten allogenen mesenchymalen Stammzellen (MSCs) aus dem Knochenmark zur Behandlung von Hautwunden in einem caninen Modell evaluiert.

**Hypothese**

Die topische allogene MSC Transplantation kann den Wundverschluss experimenteller Hautwunden (Brandwunden Grad III) beschleunigen und die lokale Entzündung mildern.

**Tiere**

Erwachsene gesunde Beagles (*n*=10; 3-6 Jahre alt; 7,2-13,1kg) wurden in der Studie verwendet.

**Methoden**

Es wurden Hautwunden 3. Grades am Rücken der gesunden Beagles verursacht und allogene MSCs intradermal injiziert. Die Schnelligkeit des Wundverschlusses und das Ausmaß der Kollagenproduktion wurden histologisch mittels Hämatoxylin und Eosin Färbung und Trichromfärbung untersucht. Das Ausmaß der zellulären Proliferation und der Angiogenese wurde mittels Immunzytochemie unter Verwendung von Proliferating*-*Cell*-*Nuclear*-*Antigen, Vimentin und Alpha Smooth Muscle Actin-spezifischen Antikörpern evaluiert. Die lokale Exprimierung von mRNA von Interleukin-2, Interferon-γ, Basic Fibroblasten Wachstumsfaktor und Matrix Metalloproteinase-2 wurde mittels RT PCR evaluiert.

**Ergebnisse**

Im Vergleich zu den Wunden, die nur mit dem Trägermedium behandelt worden waren, zeigten die mit MSC-behandelten Wunden einen rascheren Verschluss und eine erhöhte Kollagensynthese, zelluläre Proliferation und Angiogenese. Darüber hinaus zeigten MSC-behandelte Wunden eine verminderte Exprimierung pro-entzündlicher Zytokine (Interleukin-2 und Interferon-γ) und Wundheilungsfaktoren (Basic Fibroblasten Wachstumsfaktor und Matrix Metalloproteinase-2).

**Schlussfolgerung und klinische Bedeutung**

Die topische Transplantation von MSCs resultiert in parakrinen Effekten auf die zelluläre Proliferation und Angiogenese sowie die Modulierung lokaler mRNA Exprimierung verschiedener Faktoren, die mit der Wundheilung der Haut im Zusammenhang stehen.

**摘要:**

**背景**

以成体干细胞的生物工程法用于组织修复治疗已被广泛研究。将骨髓培养得到的外源性间充质干细胞(MSCs)用于犬模型上治疗皮肤创伤,我们来评估其临床价值和安全性。

**假设**

局部外源性MSC移植能使试验的皮肤创口全层愈合加快,并使局部炎症减轻。

**动物**

研究成年健康比格犬(*n* = 10;3–6 岁;7.2–13.1 kg)。

**方法**

在健康的比格犬背部创造全层皮肤创口,皮内注射外源性MSCs。使用苏木精和伊红染色法和三色染色法,从组织结构上分析伤口愈合率和胶原产生的程度。通过免疫细胞化学使用增殖细胞核抗原、维生素和α-平滑肌肌动蛋白-特定抗体,评估细胞增殖和血管生成程度。通过RT PCR评估白介素-2、γ-干扰素、基纤维母细胞生长因子和基质金属蛋白酶-2局部mRNA表达等级。

**结果**

与媒介物治疗创口相比,MSC治疗创口显示出更快的创口愈合,以及胶原合成、细胞增殖和血管生成增加。 此外,MSC治疗创口显示出炎性细胞因子(白介素-2和γ-干扰素)和与创口愈合相关因子(碱性成纤维细胞生长因子和基质金属蛋白酶-2)表达减少。

**结论和临床价值**

局部移植MSCs造成了对细胞增殖和血管生成的旁分泌作用,也同样引起几个与皮肤创伤愈合相关因子局部mRNA表达的调节。

**要約:**

**背景**

成熟した幹細胞は組織修復療法に対して生物工学的なアプローチで広く研究されている。著者らは培養した骨髄由来の同種異系の間葉系肝細胞(MSCs)を犬の皮膚創傷の治療に応用し、臨床的な価値と安全性を評価した。

**仮説**

同種異系MSCの局所への移植は実験的な皮膚全層創傷の閉鎖と炎症の軽減を加速する可能性がある。

**供与動物**

成熟した健常ビーグル犬(n = 10; 3–6 歳齢; 7.2–13.1 kg)を用いた。

**方法**

健常ビーグルの背部に皮膚全層潰瘍を作成し、同種異系MSCを皮内に注射した。創傷閉鎖率とコラーゲン産生度をヘマトキシリン染色トリクローム染色を用いて組織学的に解析した。細胞増殖度と血管新生は核内増殖抗原−、ビメンチン−、α−平滑筋アクチン−特異的抗体を用いて免疫組織化学染色で評価した。局所のインターロイキン−2、インターフェロンγ、塩基性線維芽細胞増殖因子とマトリックスメタロプロテアーゼ2のmRNA発現レベルをRT-PCRで評価した。

**結果**

賦形剤のみで治療した創傷と比較し、MSCで治療した創傷はより早期に閉鎖し、コラーゲン合成の増加、細胞増殖や血管新生を示した。さらに、MSCで治療した創傷は炎症後サイトカイン(インターロイキン−2とインターフェロン−γ)と創傷治癒関連因子(塩基性線維芽細胞増殖因子とマトリックスメタロプロテアーゼ−2)の発現が減少していた。

**結論と臨床的な重要性**

MSCの局所への移植により細胞増殖と血管新生に対してはパラクライン効果を示すのと同時に,皮膚創傷治癒に関連する様々な因子の局所mRNA発現を調節した。

## Introduction

Normal skin depends on the pool of adult stem cells, such as epidermal skin stem cells and bone marrow (BM) stem cells, for its renewal and maintenance.^1^ After skin injury, an elaborate series of events progresses, involving haemostasis, inflammation, cellular proliferation, angiogenesis and extracellular matrix production.^2^–^5^ Cytokines, chemokines and growth factors, produced from inflammatory cells, skin progenitor cells, fibroblasts and BM-derived progenitor cells, are crucial in this process.^2^,^6^,^7^ In pathological conditions, such as diabetes mellitus, chronic steroid therapy or immune suppression, and following large injuries or burns, wound healing is delayed or not completed.^8^,^9^ Nonhealing or chronic skin wounds have many contributory factors, including impairment in the production of cytokines and reduced angiogenesis.^3^,^7^,^10^–^12^ In patients with BM depletion or extensive skin tissue destruction, the host adult stem cell pool cannot function effectively, resulting in delayed healing or nonhealing skin wounds.^7^ With the advancement of bioengineering applications, biosynthetic skin equivalents using biomaterial or cultured keratinocytes or fibroblasts have shown effectiveness in treating delayed wound healing.^13^,^14^ Given that mesenchymal stem cells (MSCs) exhibit multipotency and regenerative activity, these cells were proposed as a new alternative cellular source of skin equivalents. Several previous studies of MSC transplantation in animal models and human patients have demonstrated the therapeutic effects of more rapid wound healing and improved dermal regeneration.^2^,^3^,^5^–^7^,^11^,^12^,^15^–^18^

Autologous MSC transplantation is time consuming from cell collection to application and requires the patient to have normal BM function. In contrast, allogenic MSC transplantation has the advantage of prompt preparation and can be applied independently of the health status of the patient. However, allogenic cell transplantation has the risk of graft rejection, and the failure of allogenic cell transplantation has also been reported.^14^ Therefore, identification of the positive and adverse effects of allogenic MSC transplantation for skin wound healing using a canine model is needed prior to the initiation of human clinical trials. The purpose of this study was to investigate the effects of intradermal (i.d.) MSC transplantation for wound closure and skin regeneration using a canine cutaneous wound model.

## Materials and methods

### Canine cutaneous wound model

This study received ethical approval, and all procedures dealing with animal care, handling and sampling were approved by the Institutional Animal Care and Use Committee of Konkuk University (approval no. KU08027).

**Figure 1:**
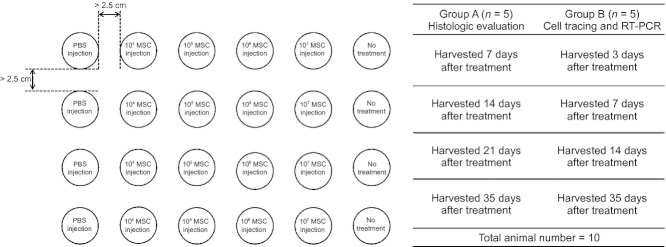
Schematic representation of experimental wound creation and treatment regimen for each wound. Abbreviations: MSC, mesenchymal stem cell; and PBS, phosphate-buffered saline.

Healthy beagle dogs (*n* = 10; 3–6 years old; 7.2–13.1 kg) were used for this study. After overnight fasting, the experimental dogs were premedicated with subcutaneous atropine sulfate (0.02 mg/kg; Jeil Pharm, Seoul, Korea) and intravenous cefazolin sodium (22 mg/kg; Chong Kun Dang Pharm, Seoul, Korea). Under general anaesthesia using intramuscular (i.m.) tiletamine–zolazepam (10 mg/kg; Zoletil; Virbac, Carros cedex, France), 24 full-thickness circular wounds of 6 mm in diameter were created on the back of each dog using disposable dermal biopsy punches. The wounds were at least 2.5 cm apart (Figure 1). After creation of the wounds, tramadol (5 mg/kg; Tridol; Yuhan Corporation, Seoul, Korea) was administered i.m. for pain control. The day on which the wounds were created was designated day 0.

### Isolation and expansion of canine allogenic BM-derived MSCs

The procedure for the preparation of canine allogenic MSCs was described in a previous report.^19^ Briefly, fresh BM was aspirated from the iliac crest of healthy donor dogs under general anaesthesia, and MSCs were isolated and cultured. Subcultured MSCs were analysed by flow cytometry and considered to be mesenchymal cells on the basis of being positive for the cell surface antigens CD9 and CD44 and negative for the haematopoietic markers CD34 and CD45.^20^–^22^ The cells from the second passage were frozen and kept at −196°C in a liquid nitrogen tank and thawed immediately prior to use. The thawed MSCs were labelled with a fluorescent cell-tracing dye using a carboxyfluorescein diacetate-succinimidyl ester (CFDA-SE) cell tracer kit (Vibrant CFDA-SE; Life Technologies, Carlsbad, CA, USA) according to the manufacturer's instructions. Finally, multiple solutions containing the desired number of labelled MSCs diluted in phosphate-buffered saline (PBS) were obtained.

### Intradermal MSC transplantation

Twenty-four hours after wound creation, five different solutions, namely 1 × 10^4^, 1 × 10^5^, 1 × 10^6^ and 1 × 10^7^ MSCs/300 μL of PBS and the same volume of PBS as a vehicle control, were injected i.d. into four wounds (100 μL for direct injection into the wound bed and 200 μL for injections spaced in a radial pattern around the wound edge). The four remaining wounds on each dog were left untreated to monitor the progress of healing. A total of 10 dogs received a set of these treatments, comprising five animals for histological analysis and the other five for cell tracing and molecular analysis (Figure 1). The treatment regimen for each dog is described in Table 1. The experimental dogs were not given any medication for immune suppression. Monitoring of the general condition and wound healing of the experimental dogs was performed daily, and the wounds were photographed using a digital camera (Power shop G9; Canon, Tokyo, Japan).

**Table 1:** The primer sets used in the RT-PCR of factors associated with wound healing

Factor	Primer type	Oligonucleotides (5′–3′)	Product size (bp)
GAPDH	Forward	GGTCACCAGGGCTGCTTT	209
	Reverse	ATTTGATGTTGGCGGGAT	
KGF-1	Forward	ATGAACACCCGGAGCACTAC	172
	Reverse	GGGCTGGAACAGTTCACATT	
bFGF	Forward	AGCAGAAGAGAGAGGCGTTG	213
	Reverse	ACTGCCCAGTTCGTTTCAGT	
MMP-2	Forward	ATGGCAAATACGGCTTCTGC	288
	Reverse	TGCAGCTCTCATGCTTGTTG	
IL-2	Forward	CCTCAACTCCTGCCACAATGT	70
	Reverse	TGCGACAAGTACAAGCGTCAGT	
IFN-γ	Forward	GCATTCCAGTTGCTGCCTACT	138
	Reverse	ACCAGGCATGAGAAGAAATGCT	

Abbreviations: bFGF, basic fibroblast growth factor; GAPDH, glyceraldehyde 3-phosphate dehydrogenase; IL-2, interleukin-2; INF-γ, interferon-γ; KGF-1, keratinocyte growth factor-1; and MMP-2, matrix metalloproteinase-2.

### Skin biopsy and histological analysis

On days 7, 14, 21 and 35, five experimental dogs were sedated using i.m. medetomidine (40 μg/kg; Domitor; Pfizer Inc., New York, NY, USA), and wounds were harvested using 8 mm dermal biopsy punches. After skin harvesting, tramadol was administered. The samples were bisected along the widest line of the wound, then fixed and paraffin embedded, sectioned into 4-μm-thick slices and prepared for histology. All stained slides were examined under a light microscope (BK51; Olympus, Tokyo, Japan), and digital photographs of the sections were taken using imaging software (DP Controller and DP Manager; Olympus). The photographs were quantitatively analysed using an image analysis program (i-solution; IMT i-solution Inc., Vancouver, BC, Canada). All measurements were performed by two investigators blinded to the treatment procedure.

For comparison of the rate of re-epithelialization on day 7, tissue sections were stained with haematoxylin and eosin, and the epithelial gap (EG) was measured, which was defined as the distance between the advancing opposite edges of epithelial cell migration.^10^,^19^ To assess the degree of collagen synthesis, Masson's trichrome stain was used. Over five random high-power fields (hpfs; ×400) in the granulation tissue, the area of collagen deposition was measured, and the percentage collagen content of the granulation tissue was calculated as the area of collagen/area of granulation tissue × 100.

### Immunohistochemical analysis of cellular proliferation and angiogenesis

To identify the effects of MSC transplantation on cellular proliferation and angiogenesis, an immunohistochemical evaluation was performed using the 1 × 10^7^ MSC-treated wounds and PBS-treated wounds harvested on days 7, 14 and 21. The concentration of 1 × 10^7^ was chosen because the 1 × 10^7^ MSC-treated wounds showed the smallest EG and the greatest collagen deposition. Proliferating cell nuclear antigen (*PCNA*), vimentin and α-smooth muscle actin (*α-SMA*) were detected using specific monoclonal antibodies (using 1:400 dilutions, for *PCNA* and *α-SMA*; Dako, Glostrup, Denmark; and using a 1:100 dilution for vimentin; Labvision/Neomarker, Fremont, CA, USA). The tissue sections were deparaffinized and hydrated, and sections for *PCNA* and vimentin immunostaining were pretreated for antigen retrieval using a pressure cooker in 10 mm citrate buffer. Endogenous peroxidases were quenched with 3% hydrogen peroxide for 15 min. Nonspecific antibody binding was blocked by incubation with a protein blocker (Dako). The sections were incubated with the primary antibodies or bovine serum albumin as a negative control for 30 min at 45°C, then incubated with horseradish peroxidase−conjugated anti-mouse and anti-rabbit immunoglobulin (Dako) for 15 min at 45°C, followed by a 5 min incubation with 3,3′-diaminobenzidine (DAB; Dako). Finally, the sections were counterstained with Gill's haematoxylin, mounted and examined under a light microscope. High-power images were used to quantify the number of DAB-positive cells or the area occupied by DAB-positive cells.

The degree of epidermal cell proliferation was assessed by the number of proliferating cells in the newly formed epidermis. Three hpfs of the sections were selected randomly, the number of *PCNA*-positive cells in the epidermis was counted, and the number of these cells per 0.1 mm^2^ of epidermis was calculated. The number of proliferating cells in the granulation tissue was analysed in the same manner. To assess the cellularity of mesenchymal origin, the area occupied by vimentin-positive signals was measured, and the percentage of the vimentin-positive area in the granulation tissue was calculated. To analyse the degree of angiogenesis, the number of blood vessels demonstrating *α-SMA*-positive lumens in five random hpfs was counted. All measurements were repeated by two investigators, and the average value was used for analysis.

### Tracing of transplanted MSCs in wound tissues

On days 3, 7 and 14 after cell transplantation, 1 × 10^7^ MSC-treated wounds and PBS-treated wounds were harvested from the other five dogs using 8 mm biopsy punches under sedation. Tramadol was administered for pain relief. Harvested wound tissues were embedded in the optimum cutting temperature (OTC) compound, and slides of the 5-μm-thick cryosections were made. The slides were examined under a fluorescence microscope (BK51). Demonstration of transplanted MSCs was performed by detection of CFDA-SE prelabelled cells in tissue slides. Positive fluorescence signals having cell morphology were regarded as the retained MSCs.

### Analysis of mRNA expression in wounded skin by RT-PCR

Local expression of wound healing-related factors was evaluated in 1 × 10^7^ MSC-treated wounds and PBS-treated wounds on days 3, 7 and 14. Harvested tissues were immediately frozen and, following homogenization, total RNA was extracted using TRIzol (Invitrogen, Carlsbad, CA, USA), according to the manufacturer's instructions. Then, RT-PCR was performed (MJ mini; Bio-Rad, Tokyo, Japan) using canine-specific primers to evaluate the expression of basic fibroblast growth factor (*bFGF*), keratinocyte growth factor-1 (*KGF-1*), interleukin-2 (*IL-2*), interferon-γ (*IFN-γ*) and matrix metalloproteinase-2 (*MMP-2*). The PCR conditions were as follows: 30 s at 94°C for denaturation, followed by 30 cycles of denaturation for 30 s at 94°C, annealing for 30 s at 58°C, and extension for 30 s at 72°C. The primer sequences are listed in Table 1. Normalization of the samples was accomplished using the housekeeping gene glyceraldehyde-3-phosphate dehydrogenase (*GAPDH*) after testing for stable expression in skin samples. The bands obtained from the PCR products were measured by densitometry using an image analysis program (Gene tools; Syngene, Frederick, MD, USA). The relative mRNA expression was calculated as the ratio of the density of each mRNA to the density of *GAPDH* mRNA.

### Statistical analysis

Statistical analyses were performed using the Statistical Package for the Social Sciences (spss version 12.0; SPSS Inc., IBM Corporation, Endicott, NY, USA). Nonparametric Kruskal–Wallis tests were used to compare the EG and collagen deposition among five different wounds. Mann–Whitney *U*-tests were used to assess the immunohistochemistry and RT-PCR results between the 1 × 10^7^ MSC-treated and PBS-treated wounds. Spearman rank correlations were used to analyse the relationship between the number of transplanted MSCs and the EG or collagen deposition. A value of *P* < 0.05 was considered statistically significant.

## Results

### Canine cutaneous wound healing process after allogenic MSC transplantation

All experimental dogs maintained a normal body condition, appetite, activity, body temperature and body weight throughout the entire experimental period. All wounds healed without severe wound infection, and typical scars formed at the site of each wound.

### Effects of MSCs on epithelial regeneration and wound closure

The histological results on day 7 showed epithelial regeneration, and the mean EGs of all MSC-treated wounds (2.33 ± 0.33 mm) were significantly smaller than those of the PBS-treated wounds (2.83 ± 0.15 mm; *P* < 0.05), although there was no significant difference among the MSC-treated wounds (Table 2). The EGs showed a tendency to decrease as the number of transplanted MSCs increased, but there was no statistically significant negative correlation in these values.

**Table 2:** Comparison of the measured epithelial gap between wounds on day 7

PBS	1 × 10^4^ MSCs	1 × 10^5^ MSCs	1 × 10^6^ MSCs	1 × 10^7^ MSCs
2.83 ± 0.15	2.46 ± 0.32*	2.45 ± 0.17*	2.29 ± 0.34*	2.10 ± 0.30*

All data are expressed in millimetres as means ± SD. Abbreviations: MSCs, mesenchymal stem cells; and PBS, phosphate-buffered saline.

* *P* < 0.05 versus PBS-treated wound, nonparametric Kruskal–Wallis test.

### Effects of MSCs on collagen synthesis in granulation tissue

**Figure 2:**
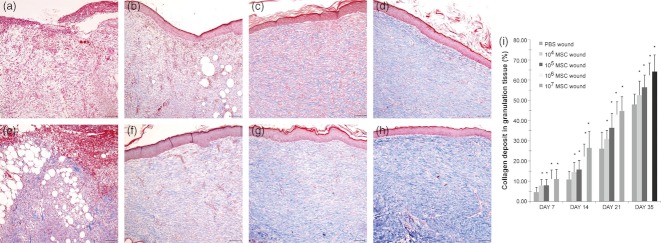
Collagen deposition in granulation tissue at day 7 (a,e), 14 (b,f), 21 (c,g) and 35 (d,h). The 1 × 10^7^ MSC-treated wounds (e–h) showed thicker and denser collagen deposition in the granulation tissue than the PBS-treated wounds (a–d). Masson's trichrome stain, original magnification ×200. (i) Comparison of the percentage of collagen fibres in granulation tissue of the wounds. All MSC-treated wounds showed a statistically significant increase in collagen synthesis compared with the PBS-treated wounds. *P < 0.05 versus PBS-treated wounds.

In the Masson's trichrome-stained sections, all MSC-treated wounds showed higher blue staining densities than the PBS-treated wounds (*P* < 0.05; Figure 2). The degree of new collagen deposition was highest in the 1 × 10^7^ MSC-treated wounds and demonstrated a trend to increase as the number of transplanted MSCs increased, although no significant positive correlation was demonstrated.

### Persistence of transplanted MSCs in the wounded skin

**Figure 3:**
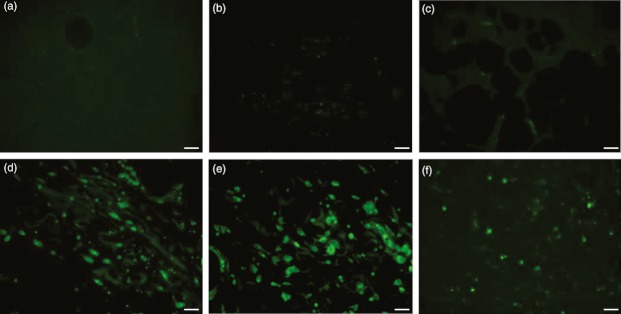
Identification of transplanted MSCs in wound bed. The PBS-treated wounds at day 3 (a), 7 (b) and 14 (c) showed no fluorescence-positive cells. Carboxyfluorescein diacetate-succinimidyl ester (CFDA-SE) prelabelled transplanted MSCs were identified as fluorescence-positive cells in the wound base of the MSC-treated wounds at day 3 (d), 7 (e) and 14 (f). The number of fluorescence-positive cells was limited in the wound bed, and the number of cells abruptly decreased as time passed. Scale bars represent 25 μm.

In the cryosections of the MSC-treated wounds, fluorescence-positive cells were detected in the wound base at day 3, but were not identified in the epidermal region (Figure 3d). The CFDA-SE cells were clumped in what was suspected to be the injection area. On day 7, sparse fluorescence-positive signals were revealed in the wound base, although the number of positive signals was significantly decreased (Figure 3e). The fluorescence-positive cells were seldom detected as time progressed, and there was little signal detected in the cryosections on day 14 (Figure 3f).

### Effects of MSCs on cellular proliferation

**Figure 4:**
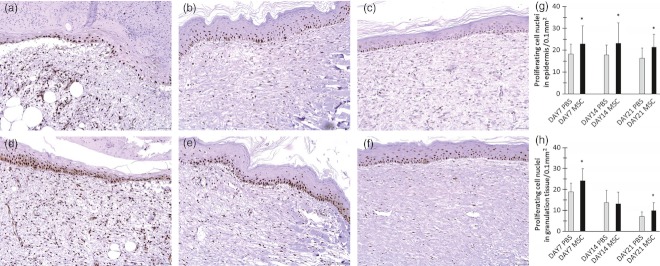
Immunostaining of proliferating cell nuclear antigen (PCNA) at day 7 (a,d), 14 (b,e) and 21 (c,f). The 1 × 10^7^ MSC-treated wounds (d–f) showed a higher number of PCNA-positive nuclei in both the newly formed epidermis and the granulation tissue compared with the PBS-treated wounds (a–c). Original magnification ×200. (g,h) Comparison of cellular proliferation in epidermis (g) and granulation tissue (h). In the newly formed epidermis of 1 × 10^7^ MSC-treated wounds, PCNA-positive cells were continuously identified until day 21 postwounding. *P < 0.05 versus PBS-treated wounds.

The number of PCNA-positive nuclei in the epidermis of the MSC-treated wounds (22.9/0.1 mm^2^) was significantly higher than that of the PBS-treated wounds (18.3/0.1 mm^2^; *P* = 0.043) on day 7 (Figure 4). Additionally, in the granulation tissue, the MSC-treated wounds showed significantly more positive nuclei (24.2 versus 19.0/0.1 mm^2^; *P* = 0.007) on days 7 and 21 (9.9 versus 7.3/0.1 mm^2^; *P* = 0.004; Figure 4).

**Figure 5:**
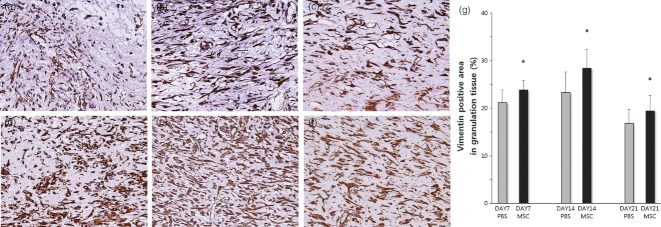
Comparison of the cellularity of mesenchymal origin in the granulation tissue at day 7 (a,d), 14 (b,d) and 21 (c,f). The 1 × 10^7^ MSC-treated wounds (d–f) had more vimentin-positive cells compared with the PBS-treated wounds (a–c). Original magnification ×200. (g) Statistical analysis demonstrated that the degree of distribution of mesenchymal cells was higher in the 1 × 10^7^ MSC-treated wounds. *P < 0.05 versus PBS-treated wounds.

The ratio of the vimentin-positive stained area to the granulation tissue per unit area was higher in the MSC-treated wounds (23.80%) than in the PBS-treated wounds (21.13%; *P* = 0.001) on day 7. The increase in cells of mesenchymal origin in the MSC-treated wounds was maintained until day 21 (Figure 5).

### Effects of MSCs on angiogenesis

The MSC-treated wounds showed greater blood vessel density than the PBS-treated wounds during the early wound healing process. On day 7, the MSC-treated wounds showed greater wound vascularity (8.5 blood vessels/hpf) than the PBS-treated wounds (3.7 blood vessels/hpf; *P* < 0.05). On day 14, the number of blood vessels declined and there was no significant difference in the vascularity of the MSC-treated wounds and the PBS-treated wounds (Table 3).

**Table 3:** The number of blood vessels in the granulation tissue of each type of wound

Time point	PBS-treated wounds	MSC-treated wounds
Day 7	3.7 ± 2.46/hpf	8.5 ± 2.70/hpf*
Day 14	9.1 ± 4.96/hpf	12.9 ± 5.08/hpf*
Day 21	4.6 ± 1.88/hpf	5.05 ± 2.36/hpf

All data are expressed as means ± SD. Abbreviations: hpf, high-power field; MSC, mesenchymal stem cell; and PBS, phosphate-buffered saline.

* *P* < 0.05 versus PBS-treated wound, nonparametric Kruskal–Wallis test.

### Expression of wound healing-related factors in wounded tissue

**Figure 6:**
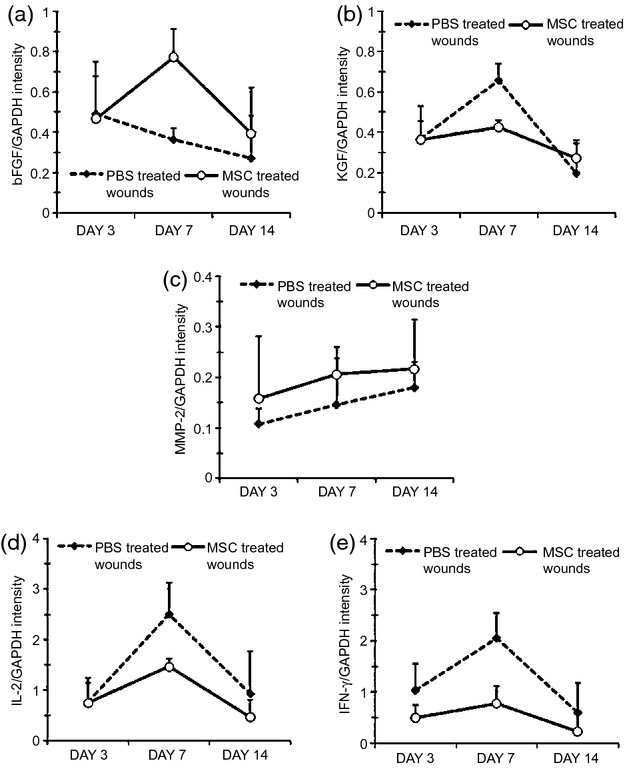
Quantification of mRNA expression of cytokines in wound tissue at day 3, 7 and 14. (a,b) The expression of basic fibroblast growth factor (bFGF) and keratinocyte growth factor-1 (KGF-1) showed a contrary trend of change at day 7 postwounding; bFGF was higher in the 1 × 10^7^ MSC-treated wounds, whereas KGF-1 was higher in the PBS-treated wounds. (c) The expression of matrix metalloproteinase-2 (MMP-2) increased slightly as time passed and was higher in the 1 × 10^7^ MSC-treated wounds than in the PBS-treated wounds (d,e). Expression of interleukin-2 (IL-2) and interferon-γ (INF-γ) increased at day 7 postwounding and then decreased, and the 1 × 10^7^ MSC-treated wounds showed lower and not significantly increased expression compared with the PBS-treated wounds.

The expression of wound healing-related factors in the 1 × 10^7^ MSC-treated wounds and the PBS-treated wounds was measured at the mRNA level by quantitative RT-PCR. On day 7, expression of *bFGF* was increased in the MSC-treated wounds but was decreased in the PBS-treated wounds. The expression of *bFGF* in the MSC-treated wounds was decreased on day 14. Meanwhile, expression of *KGF-1* was higher in the PBS-treated wounds than in the MSC-treated wounds. Expression of *KGF-1* also declined on day 14 postwounding in both types of wounds. Expression of *MMP-2* demonstrated a slight increase over time, and the MSC-treated wounds showed increased *MMP-2* expression from day 3 to 14. Expression of *IL-2* and *IFN-γ* showed a similar change, increasing by day 7 and decreasing by day 14. The MSC-treated wounds maintained lower IL-2 and *IFN-γ* expression from day 3 to 14 postwounding relative to the PBS-treated wounds (Figure 6).

## Discussion

In this study, MSC-treated wounds showed more rapid wound closure, and this was presumed to be due to MSCs promoting re-epithelialization. Faster epithelialization, following BM-derived stem cell transplantation for cutaneous wound healing, has been demonstrated in previous studies in other species.^16^,^18^,^22^,^23^ The actions of MSCs may include promotion of keratinocyte migration and proliferation. This was demonstrated in the present study, in that the proliferation of keratinocytes was more active in MSC-treated wounds as determined by immunohistochemistry to detect *PCNA*.

In the trichrome-stained sections, increased collagen deposition in MSC-treated wounds was demonstrated, which indicated the promotion of fibroblast proliferation, as shown by the vimentin and *PCNA* immunohistochemistry results. Other studies in other species have shown that MSCs promote fibroblast proliferation, resulting in intensified granulation tissue formation.^4^,^11^,^17^,^22^,^24^,^25^ The accumulation of large collagen fibres is likely to increase the wound strength.^2^

Apart from fibroblast proliferation, the formation of new blood vessels is necessary to maintain granulation tissue.^2^,^22^ In the present study, granulation tissue angiogenesis was more active in MSC-treated wounds, as demonstrated by immunostaining with *α-SMA*, which is in agreement with previous studies.^3^ The microfilament protein *α-SMA* is a marker of mature blood vessels.^26^ Several factors stimulate angiogenesis, and MSCs can attract macrophages and vascular endothelial cells into wounded tissue by releasing various angiogenic factors.^3^,^17^,^18^ Among those factors, *bFGF* has a promoting effect on the growth and migration of endothelial cells,^2^,^3^,^8^ while *MMP-2*, a member of the family of matrix metalloproteinases, plays a role in degradation of the extracellular matrix, consequently facilitating endothelial cell migration.^27^ In the present study, the mRNA expression of *bFGF* in MSC-treated wounds was increased. At the same time, increased mRNA expression of *MMP-2* in MSC-treated wounds was confirmed. Thus, upregulation of *bFGF* and *MMP-2* in MSC-treated wounds can be attributed partly to increased blood vessel formation.

In addition, as mentioned above, fibroblasts play a major role in wound healing.^27^ Increased expression of *bFGF* could also partly explain the promotion of fibroblast proliferation and increased production of collagen fibres. Although accumulation of larger collagen fibres increases the wound strength, excessive accumulation of collagen may induce hypertrophic scarring.^2^ Matrix metalloproteinases also control collagen degradation and play a role in remodelling of wounds to prevent extensive scar formation and fibrotic changes.^2^ Increased *MMP* in MSC-treated wounds can be favourable for improving the quality of the healed wounds. Thus, MSCs seemed to influence the degree of wound maturation. Prevention of excessive wound contraction or hypertrophic scar formation is important for maintaining normal function of the skin and its cosmetic appearance. To obtain a therapeutic strategy involving less wound contraction, dermal substitutes or biomaterial have been applied.^28^ The ability of MSCs to regulate collagen production should be investigated further to obtain more satisfactory outcomes for the healing of large skin wounds.

Damage of the skin inevitably induces local inflammation and recruits inflammatory cells, which act as sources of the cytokines that affect the wound healing process.^2^ However, massive or sustained inflammation can delay wound healing; therefore, local suppression of inflammation may be beneficial to this process. Previous studies demonstrated that MSCs can downregulate pro-inflammatory cytokines and upregulate anti-inflammatory cytokines.^29^,^30^ The present study showed that MSCs downregulated the expression of the pro-inflammatory cytokines *IL-2* and *IFN-γ*, which implies that MSCs exert a suppressive effect on local inflammation. The *IL-2* and *IFN-γ* levels do not represent the complete state of tissue inflammation. However, the results of the present study demonstrated that the MSCs exhibited a paracrine effect of decreasing postinjury inflammation. In particular, *IFN-γ* is known to impair re-epithelialization;^8^ therefore, suppression of local *IFN-γ* expression may act favourably in the wound healing process.

There has been no consensus explanation for the exact mechanism of action of MSCs on wound healing. One hypothesis involves transdifferentiation of MSCs into epithelial cells. The engrafted MSCs were seldom detected, and transdifferentiation of the MSCs into keratinocytes was not demonstrated in the present study, although several previous studies demonstrated transdifferentiation of MSCs both *in vitro*^15^,^31^,^32^ and *in vivo*.^4^,^6^,^18^,^33^ However, the results of these previous *in vivo* studies were inconsistent, and the persistence of the transplanted MSCs was very low in some studies.^16^,^34^ One previous study suggested that the proper microenvironment was essential for survival and transdifferentiation of MSCs.^34^ Recently, the use of scaffolds or hydrogel has demonstrated more epidermal transdifferentiation of MSCs than previous studies.^31^,^33^ Thus, the survival and transdifferentiation of transplanted MSCs might be influenced by both the environment of the transplanted site and complex cellular interactions.

Another hypothesis is that MSCs exert paracrine effects on wound healing, such as promoting fibroblast proliferation or exaggerating angiogenesis.^34^,^35^ Mesenchymal stem cells release significant amounts of growth factors, such as *KGF*, epidermal growth factor and transforming growth factor-β, in a hypoxic environment *in vitro*.^17^ The paracrine effect hypothesis is more widely accepted than the direct transdifferentiation hypothesis, and it is unknown whether transdifferentiated cells from MSCs have functions that are similar to those of the cells of the original skin tissue.^32^ The present study demonstrated that the local expression of wound healing-related factors and pro-inflammatory cytokines was modulated by MSCs. Transdifferentiation of transplanted MSCs might not lead to beneficial long-term results. The possibility of transdifferentiation, in other words, can introduce the potential for teratogenesis or oncogenesis. Thus, strategies aimed at maximizing the paracrine effects of MSCs may be more beneficial than those that induce transdifferentiation during the wound healing process.

In the present study, there was no statistically significant difference in the results obtained between the different concentrations MSCs that were transplanted. This finding conflicts with a previous human clinical trial,^10^ which reported a strong correlation between the cell number and the rate of ulcer healing. The study suggested that at least more than 1 × 10^6^ cells/cm^2^ of wound was needed for a significant therapeutic effect. In contrast, the present study failed to determine the therapeutic dose of topical MSC transplantation. Confirmation of the therapeutic level of cell transplantation may be crucial in clinical cell transplantation therapy, because stem cell transplantation is associated with the underlying risks of the teratogenic and oncogenic processes; therefore, the appropriate number of cells needed for efficient wound healing must be investigated further, and the appropriate number of cells may differ according to the route of delivery.

One study reported decreased engraftment in the wound site of systemically transplanted MSCs without chemo-attractants; systemic allogenic cell transplantation is not free from the risk of a host reaction leading to transplantation failure.^34^ Local delivery using i.d. or i.m. injection is convenient and may require a relatively small number of cells. Furthermore, local transplantation does not require pretransplantation radiation or chemical immunological inhibition in patients whose health status is not appropriate for immunosuppressive therapy. Recently, MSC application using amniotic membrane grafting showed effective promotion of wound healing.^12^ The use of scaffolds can provide a higher retention rate of transplanted cells, although biomaterials might stimulate the immune response of the donor.^36^,^37^ Consequently, more effective methods of MSC transplantation need to be investigated.

In conclusion, topical injection of MSCs resulted in more rapid re-epithelialization and increased collagen deposition and angiogenesis in a canine wound healing model. In addition, topically applied MSCs appeared to have a suppressive effect on local inflammation in the wounded skin, suggesting that MSCs may be applied in patients with not only large or nonhealing skin defects but also inflammatory and fibrotic skin diseases.
